# Association of blood biomarkers and autoimmunity with immune related adverse events in patients with cancer treated with immune checkpoint inhibitors

**DOI:** 10.1038/s41598-021-88307-3

**Published:** 2021-04-27

**Authors:** Despina Michailidou, Ali Raza Khaki, Maria Pia Morelli, Leonidas Diamantopoulos, Namrata Singh, Petros Grivas

**Affiliations:** 1 Division of Rheumatology, Department of Medicine, University of Washington, Seattle, WA USA; 2 Division of Medical Oncology, Department of Medicine, University of Washington, 1144 Eastlake Ave E, LG-465, Seattle, WA 98109 USA; 3 Clinical Research Division, Fred Hutchinson Cancer Research Center, 1144 Eastlake Ave E, LG-465, Seattle, WA 98109 USA; 4 Seattle Cancer Care Alliance, 1144 Eastlake Ave E, LG-465, Seattle, WA 98109 USA; 5 Division of Oncology, Department of Medicine, Stanford University, Palo Alto, CA USA; 6 Gastrointestinal Medical Oncology, The University of Texas, MD Anderson Comprehensive Cancer Center, Houston, TX USA; 7 Department of Medicine, University of Pittsburgh, Pittsburgh, PA USA

**Keywords:** Cancer, Immunology, Oncology, Rheumatology

## Abstract

Patients with cancer treated with immune checkpoint inhibitors (ICIs) develop immune related adverse events (irAEs), however biomarkers are lacking. We hypothesized that clinicopathologic and laboratory factors would be associated with irAE risk and overall survival (OS) in this population. In a retrospective study of patients treated with ICIs we collected clinicopathologic, laboratory, irAEs and outcomes data. The association between baseline blood biomarkers, clinicopathologic features and irAEs was assessed by logistic regression adjusting for age, sex, smoking, cancer type, performance status, concomitant other systemic therapy, history of autoimmune disease (AD), chronic infection and pre-existing systemic steroid use (regardless of dose). Optimal cutoff values of biomarkers were identified by recursive partitioning analysis. 470 patients were identified; 156 (33%) developed irAEs, which were associated with baseline absolute lymphocyte count > 2.6 k/ul (adjusted [a]OR: 4.30), absolute monocyte count > 0.29 k/ul (aOR: 2.34) and platelet count > 145 k/ul (aOR: 2.23), neutrophil to lymphocyte ratio (NLR) ≤ 5.3 (aOR: 2.07) and monocyte to lymphocyte ratio (MLR) ≤ 0.73 (aOR: 2.96), as well as platelet to lymphocyte ratio ≤ 534 (aOR: 5.05). Patients with pre-existing AD (aOR: 2.57), family history of AD (aOR: 5.98), and ICI combination (aOR: 2.00) had higher odds of irAEs. Baseline NLR ≤ 5.3 (aHR: 0.68), MLR ≤ 0.73 (aHR: 0.43), PLT > 145 (aHR: 0.48) and PLR ≤ 534 (aHR: 0.48) were associated with longer OS. irAEs were associated with autoimmune history, ICI combination and baseline laboratory measurements. Lower NLR, MLR and PLR may have favorable prognostic value. Our hypothesis-generating findings require validation in larger prospective studies.

## Introduction

Immune checkpoint inhibitors (ICIs) have altered the therapeutic landscape of immunotherapy in cancer^1^. ICIs block regulatory T-cells immunosuppression by blocking intrinsic down-regulators of immunity, such as cytotoxic T-lymphocyte antigen 4 (CTLA-4) and programmed cell death 1 (PD-1) or its ligand, programmed cell death ligand 1 (PD-L1)^2^. This inhibition can lead to an effective anti-tumor immune response but can trigger significant immune related adverse events (irAEs)^3^ causing significant morbidity, mortality and increasing costs^4–6^.

The reported incidence rate of irAEs of any grade is about 60–70% in patients treated with ipilimumab^7^, whereas in patients receiving anti-PD-(L)1 agents seems lower^8^. The incidence of fatal irAEs is estimated to be from 0.3 to 1.3%^5^. Target sites of irAEs can be any human tissue including, but not limited to, gut, skin, lungs, and endocrine glands^9^. Rheumatologic irAEs have also been reported^10, 11^ and require the collaborative, inter-disciplinary management by rheumatologists and oncologists. Interestingly, initial clinical trials with ICIs excluded patients with preexisting active rheumatologic or other autoimmune disease (AD) due to concerns of potential exacerbation^12^. Oncologists frequently face the clinical dilemma on whether or not to administer ICIs to patients with pre-existing AD, as 20–40% of them develop unrelated irAEs or exacerbation of their underlying disease^1, 13, 14^. Further assessment of real-world incidence of irAEs and of putative associated risk factors can enrich the literature and generate hypothesis for future studies, as well as inform clinical discussions. Moreover, the clinical relevance of family history of AD, its association with irAEs and impact on clinical decision making requires further evaluation.

Due to risk of exacerbation of autoimmune phenomena causing morbidity and mortality, there is an urgent unmet need to identify additional demographic, clinicopathologic and laboratory features that may be associated with higher irAE risk, helping oncologists and patients carefully weigh the benefit/risk ratio of ICIs in practice.

The objectives for this retrospective cohort study of patients who received ICIs for either solid tumors or hematological malignancies were to: (1) explore whether there is an association between irAEs and a. absolute blood cell counts and their ratios at baseline; b. select clinicopathologic features; (2) to evaluate the potential association between baseline blood count biomarkers and overall survival (OS). We hypothesized that baseline blood counts and select clinicopathologic features would be associated with the risk of irAEs and influence OS of patients with cancer treated with ICIs.

## Methods

### Study population

Patients were included from an IRB-approved retrospective observational cohort study at the University of Washington and Seattle Cancer Care Alliance (SCCA) in Seattle, WA (IRB ID: STUDY00008393). Patients who initiated therapy with anti-PD-1 (nivolumab, pembrolizumab, cemiplimab), anti-PD-L1 (atezolizumab, durvalumab, avelumab), or anti-CTLA-4 (ipilimumab, tremelimumab) ICIs between January 2018 and October 2018 were included in the study. We used analytical search algorithms to identify all consecutive patients with new ICI initiation during this time period.

### Patient and public involvement

The authors declare no direct patient and public involvement in study design.

### Clinical and laboratory assessment

Data was abstracted using REDCap data collection tools hosted by the Institute of Translational Health Sciences (ITHS)^15^. Data abstracted included demographics, any personal and/or family history of AD, personal history of chronic infection, smoking history, laboratory values, Eastern Cooperative Oncology Group performance status (ECOG PS), sites of metastatic disease at time of ICI initiation, whether concomitant administration of ICIs or other systemic therapy was given, pre-ICI systemic steroid use (regardless of dose), date of irAE development, site/organ of irAE, vital status, and date of death or last follow up. Diagnosis of irAE was defined as new onset adverse event that patients developed after ICI initiation and was determined by the treating provider to be attributed to ICI in the absence of other etiologies based on consistent chart review. Only clinically significant irAEs that required pharmacological intervention (i.e. steroids given in any dose/route and/or other immunosuppressive therapy, new hormone replacement therapy), and/or hospitalization were recorded. Chronic infection was defined as history of human immunodeficiency virus (HIV), hepatitis C (HCV), hepatitis B (HBV), or tuberculosis (bacterial infections on antibiotic were not included in this definition). Baseline laboratory parameters recorded included absolute neutrophil count (ANC), absolute lymphocyte count (ALC), absolute monocyte count (AMC), and platelet count (PLT). Neutrophil to lymphocyte ratio (NLR), monocyte to lymphocyte ratio (MLR) and platelet to lymphocyte ratio (PLR) were also calculated.

### Statistical analysis

We independently evaluated whether baseline blood counts, personal or family history of AD, treatment with combination ICI or history of chronic infection were associated with irAE development using multivariable logistic regression. For all multivariable models, we a priori adjusted for age, sex, smoking history, cancer type (hematological vs solid malignancy), ECOG PS, concomitant other systemic therapy, personal or family history of AD, personal history of chronic infection and baseline (pre-ICI) use of systemic steroids (regardless of dose). The results were reported using odds ratio (OR) with 95% confidence interval (CI).

In addition to testing the lab values on a continuous scale, we used the recursive partitioning analysis (RPA)^16^ to identify the optimal cut-off values that may be associated with irAE development for absolute cell counts and ratios. Once identified, we evaluated the association between these cut-off values and irAE development using similar multivariable models as stated above.

We also performed an exploratory analysis evaluating the potential association between our exposures of interest (baseline laboratory values, personal or family history of AD, personal history of chronic infection and concomitant ICI) and overall survival (OS). OS was calculated from the first day of treatment with ICI to death of any cause. Patients who did not die were censored at the date of last follow-up. We used the Kaplan–Meier method to estimate OS, and multivariable Cox regression to compare OS. We tested lab values using the continuous scale for the OS analysis; we also tested the cut-off points derived based on irAE development for any laboratory value that had a significant association with irAE. Hazard ratios (HR) with 95% CI were reported. For all analyses, *p* values < 0.05 were considered statistically significant. Data analysis was carried out using STATA (v16.1) and R software (3.5.0).

### Ethics approval

This research was performed under an IRB-approved protocol from the Seattle Cancer Care Alliance IRB (IRB ID: STUDY00008393). All procedures performed complied with the ethical standards of the 1964 Helsinki declaration and its later amendments.

### Informed consent

Given the retrospective nature of the study, patient consent was waived by the IRB.

## Results

### Demographic characteristics

A total of 470 patients were included in our study with median age 65; median follow-up was 25 months. Patients were predominantly non-Hispanic white (87%) and men (59%). Baseline demographic features are shown in Table 1; 315 patients (67%) received monotherapy with anti-PD-1, 56 (12%) with anti-PD-L1, only 2 (< 1%) received anti-CTLA-4 monotherapy, and, 88 (19%) received concomitant ICIs.

**Table 1:** Demographic characteristics.

	N = 470
**Age, median (IQR)**	65 (56–71)
**Sex, N (%)**	
Male	275 (59)
Female	195 (41)
**Race/ethnicity, N (%)**	
Non-Hispanic White	408 (87)
Hispanic	14 (3)
Black	3 (1)
Asian	35 (7)
Other	10 (2)
**Smoking history, N (%)**^**a**^	
Never	225 (48)
Former	199 (43)
Active	44 (9)
**Cancer site, N (%)**	
Lung	130 (28)
Skin	93 (20)
Genitourinary	68 (14)
Gastrointestinal	53 (11)
Sarcoma	46 (10)
Hematological malignancy	37 (8)
Head and neck	25 (5)
Breast	7 (1)
Other	11 (2)
**ECOG performance status, N (%)**^**a**^	
0	163 (35)
1	220 (47)
2	76 (16)
3	10 (2)
**ICI given, N (%)**	
Anti-PD-1	315 (67)
Anti-PD-L1	56 (12)
Anti-CTLA-4	2 (< 1)
Multiple given concomitantly	88 (19)
Multiple given separately	9 (2)
**Other systemic therapy with ICI, N (%)**	
Yes	142 (30)
No	328 (70)
**Personal hx of AD, N (%)**	
Yes	79 (17)
No	391 (83)
**Rheumatologic AD, N (%)**	47 (10)
Rheumatoid arthritis	6
Polymyalgia rheumatic	6
Psoriasis/psoriatic arthritis	6
Retroperitoneal fibrosis	1
Sjogren’s syndrome	1
ANCA negative vasculitis	1
Undifferentiated connective tissue disease	1
Gout/pseudogout	25
**Non-rheumatologic AD, N (%)**	32 (6)
Hypothyroidism	24
Inflammatory bowel disease	2
Atopic dermatitis	1
Autoimmune thyroiditis	1
Chronic inflammatory demyelinating polyneuropathy	1
Common variable immunodeficiency	1
Interstitial lung disease	1
Multiple sclerosis	1
**Family hx of AD, N (%)**	
Yes	19 (4)
No	451 (96)
**Rheumatologic AD, N (%)**	14 (3)
Rheumatoid arthritis	3
Systemic lupus erythematosus	3
Psoriatic arthritis/psoriasis	2
Gout	4
Myositis	1
Vasculitis	1
**Non-rheumatologic AD, N (%)**	5 (1)
Multiple sclerosis	4
Inflammatory bowel disease	1
**History of chronic infection, N (%)**	
Yes	40 (9)
No	430 (91)
**Pre-ICI immunosuppression, N (%)**	
Yes	12 (3)
No	458 (97)
**Labs at ICI initiation, median (IQR)**	
ANC (× 10^3^/μl)	4.3 (3.3–6.0)
ALC (× 10^3^/μl)	1.2 (0.8–1.7)
AMC (× 10^3^/μl)	0.5 (0.4–0.7)
Platelets (× 10^9^/L)	229 (177–293)
NLR	3.6 (2.4–6.5)
MLR	0.5 (0.3–0.7)
PLR	183 (122–280)

*IQR* interquartile range, *ECOG Performance status* Eastern Cooperative Oncology Group, *ICI* immune checkpoint inhibitor, *AD* autoimmune disease, *hx* history, *Anti-PD-1* anti-programmed cell death 1, *Anti- PD-L1* anti-programmed cell death ligand 1, *Anti-CTLA-4* anti-cytotoxic T-lymphocyte antigen 4, *ANCA negative vasculitis* anti-neutrophil cytoplasmic antibody negative vasculitis, *ANC* absolute neutrophil count, *ALC* absolute lymphocyte count, *AMC* absolute monocyte count; *NLR* neutrophil to lymphocyte ratio, *MLR* monocyte to lymphocyte ratio, *PLR* platelet to lymphocyte ratio.

^**a**^2 patients with missing smoking history and 1 patient missing ECOG PS.

### Types and frequency of irAEs

Overall, 156 out of 470 patients (33%) developed irAEs. A total of 212 irAEs were recorded as 42 out of 470 (9%) of patients developed > 1 irAEs; (15% rheumatologic; 85% non-rheumatologic). The occurrence of different irAE categories is shown in Table 2. The median number of ICI doses before the onset of irAE was four (range 1–29). The most common rheumatologic irAE was inflammatory arthritis (IA) (11/31; 35%) and the most common non-rheumatologic irAE was hypothyroidism (43/181; 24%). The median number of days to the first irAE development for each of the irAEs is reported in Table 2.

**Table 2:** Summary of rheumatologic and non-rheumatologic irAEs.

	Number	%	Days to first irAE, median (range)
**All irAEs**	212	100	
**Rheumatologic irAEs**	31	15	
Inflammatory arthritis	11	5	111.5 (34–539)
Rheumatoid arthritis	4	2	9 (5–25)
Psoriatic arthritis	4	2	59 (26–242)
Polymyalgia rheumatic	3	1.4	246 (28–379)
Gout	3	1.4	68 (2–75)
Arthralgias	3	1.4	91 (28–379)
Mixed connective tissue disease	1	0.5	*
Spondylitis	1	0.5	*
Leukocytoclastic vasculitis	1	0.5	359
**Non rheumatologic irAEs**	181	85	
**Skin**	13	6	
Non specified facial rash	2	1	197.5 (197–198)
Urticaria	1	0.5	7
Pemphigoid bullous	3	1.4	449 (327–469)
Eczematous dermatitis	2	1	28 (9–126)
Lichen planus	2	1	247.5 (215–280)
Lichen sclerosus	1	0.5	531
Vitiligo	2	1	54
**Gastrointestinal**	58	27	
Colitis	29	14	126 (4–619)
Diarrhea	5	2.3	18 (5–795)
Hepatitis	18	8.5	70 (4–545)
Cholestatic liver disease	1	0.5	*
Cholangitis	1	0.5	163
Pancreatitis	4	2	118 (73–163)
**Endocrine**	72	34	
Hypothyroidism	43	20	85 (7–342)
Thyroiditis	4	2	79 (20–146)
Adrenal insufficiency	14	7	118.5 (21–342)
Type I Diabetes mellitus	2	1	173.5 (33–314)
Hypophysitis	8	4	117 (21–341)
Hypogonadism	1	0.5	341
**Pulmonary**	25	12	
Pneumonitis	25	12	57 (7–350)
**Kidney**	5	2.4	
Interstitial nephritis	5	2.4	161.5 (15–577)
**Eye**	2	1	
Uveitis	2	1	67
**ENT**	1	0.5	
Laryngitis	1	0.5	84
**Nervous system**	2	1	
Neurotoxicity	1	0.5	5
Headache	1	0.5	*
**Heart**	1	0.5	
Myocarditis	1	0.5	41
**Hematology**	1	0.5	65
Autoimmune hemolytic anemia	1	0.5	
**Other**	1	0.5	
Cytokine release syndrome	1	0.5	*

*irAEs* immune related adverse events.

*it was not the first irAE.

### Association of blood count biomarkers with irAEs

Baseline ANC, ALC, AMC and PLT were available for 441 (94%), 391 (83%), 391 (83%) and 459 (98%) out of 470 patients, respectively. Higher baseline ALC modeled as a continuous variable was the only absolute cell count that was associated with higher odds of irAEs (adjusted [a]OR: 1.47, 95% CI 1.08–2.01, p = 0.02, Table 3). In addition, baseline ALC > 2.6 k/μl was associated with higher odds of irAE occurrence (aOR: 4.30, 95% CI 1.70–10.89, p = 0.002; Table 4), indicating that patients with cancer and baseline lymphocytosis may have a greater likelihood of developing irAEs. Baseline AMC > 0.29 k/μl and baseline platelet count > 145 k/μl were also associated with higher risk of irAEs (aOR: 2.34, 95% CI 1.06–5.15, p = 0.03 and aOR: 2.23, 95% CI 1.06–4.57, p = 0.03 respectively; Table 4).

**Table 3:** Association of blood count biomarkers as continuous variables with irAEs and OS.

Blood count biomarkers	irAEs			OS		
	aOR^a^	95% CI	p-value	aHR^a^	95% CI	p-value
Absolute neutrophil count (ANC)	1.02	0.95–1.08	0.59	1.01	0.97–1.05	0.60
Absolute lymphocyte count (ALC)	1.47	1.08–2.01	0.02	0.81	0.65–1.01	0.06
Absolute monocyte count (AMC)	1.03	0.48–2.20	0.95	2.08	1.21–3.58	0.01
Platelet count (PLT)	1.00	1.00–1.00	0.34	1.00	1.00–1.00	0.13
Neutrophil:lymphocyte ratio (NLR)	0.97	0.92–1.02	0.25	1.01	0.99–1.02	0.33
Monocyte:lymphocyte ratio (MLR)	0.36	0.18–0.74	0.01	1.27	1.05–1.54	0.01
Platelet:lymphocyte ratio (PLR)	1.00	1.00–1.00	0.26	1.00	1.00–1.00	0.77

^a^Adjusted for age, sex, smoking history, cancer type (hematological malignancy vs solid tumor), ECOG PS, concomitant other systemic therapy, personal or family history of autoimmune disease, personal history of chronic infection, systemic steroid treatment at time of ICI initiation.

**Table 4:** Association between cut off values of blood count biomarkers and irAEs.

Blood count biomarkers	Cut off values	aOR^a^	95% CI	p-value
Absolute neutrophil count (ANC)	≤ 6.5	1.41	0.79–2.53	0.25
Absolute lymphocyte count (ALC)	> 2.6	4.30	1.70–10.89	0.002
Absolute monocyte count (AMC)	> 0.29	2.34	1.06–5.15	0.03
Platelet count (PLT)	> 145	2.23	1.06–4.57	0.03
Neutrophil:lymphocyte ratio (NLR)	≤ 5.3	2.07	1.20–3.58	0.01
Monocyte:lymphocyte ratio (MLR)	≤ 0.73	2.96	1.56–5.60	0.001
Platelet:lymphocyte ratio (PLR)	≤ 534	5.05	1.09–23.37	0.04

^a^Adjusted for age, sex, smoking history, cancer type (hematological malignancy vs solid tumor), ECOG PS, concomitant other systemic therapy, personal or family history of autoimmune disease, personal history of chronic infection, systemic steroid treatment at time of ICI initiation.

Among 470 patients treated with ICIs, 390 (83%) had baseline complete blood counts available for NLR, MLR and PLR calculation. Higher baseline MLR as a continuous variable (aOR: 0.36, 95% CI 0.18–0.74, p = 0.01) was associated with lower odds of irAE development (Table 3). In addition, baseline NLR ≤ 5.3 k/μl (aOR: 2.07, 95% CI 1.20–3.58, p = 0.01), MLR ≤ 0.73 k/μl (aOR: 2.96, 95% CI 1.56–5.60, p = 0.001) and PLR ≤ 534 k/μl (aOR: 5.05, 95% CI 1.09–23.37, p = 0.04) were all associated with higher odds of irAE (Table 4).

### Association of clinicopathologic features with irAEs

Personal or family history of AD, history of chronic infection, and combination ICI were investigated among all 470 patients. Both personal (aOR: 2.57, 95% CI 1.46–4.52, p = 0.001) and family history of AD (aOR: 5.98, 95% CI 2.20–16.23, p < 0.001) were significantly associated with irAEs. In addition, combination of ICIs (aOR: 2.00, 95% CI 1.17–3.43, p = 0.01) was also associated with higher odds of irAEs. Chronic infection was not associated with irAE development (aOR: 0.53, 95% CI 0.25–1.13, p = 0.10).

### Association of blood count biomarkers with OS

**Figure 1:**
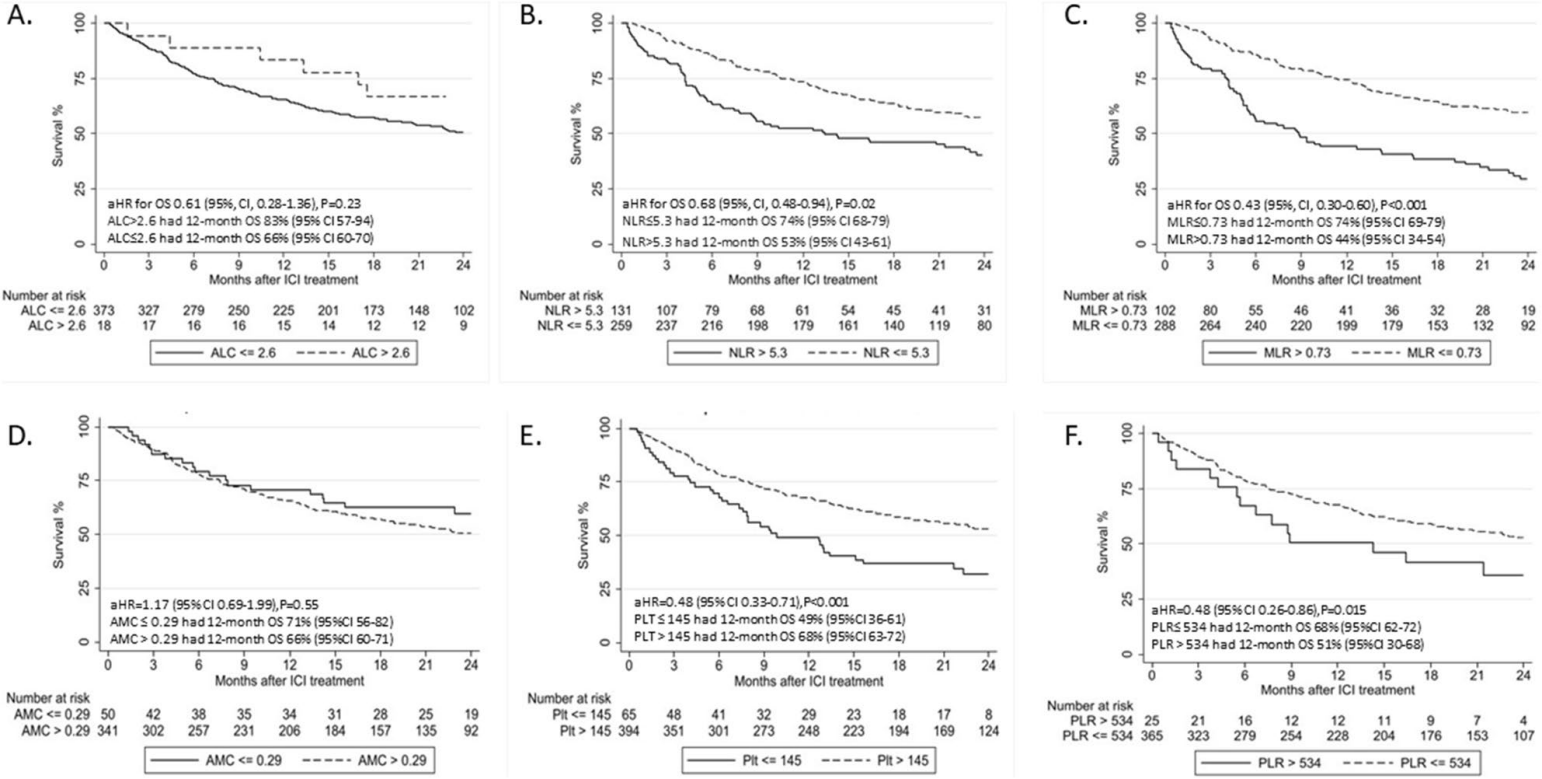
Overall survival (OS) by laboratory cutpoints. Kaplan Meier estimates of OS in patients with (**A**) absolute lymphocyte count > and ≤ 2.6k/ul, (*B*) neutrophil to lymphocyte ratio > and ≤ 5.3 (*C*) monocyte to lymphocyte ratio > and ≤ 0.73, (**D**) absolute monocyte count > and ≤ 0.29 k/ul, (**E**) platelent count > and ≤ 145 k/ul and (**F**) platelet to lymphocyte count > and ≤ 534. *aHR* adjusted hazard ratio, *ICI* immune checkpoint inhibitors, *ALC* absolute lymphocyte count, *NLR* neutrophil to lymphocyte ratio, *MLR* monocyte to lymphocyte ratio, *AMC* absolute monocyte count, *Plt* platelet count, *PLR* platelet to lymphocyte count, *OS* overall survival, *CI* confidence interval.

In an exploratory analysis, we evaluated the association between baseline absolute cell counts (and the cut-off points derived using RPA in association to irAE development) and OS. Multivariable Cox regression analysis showed that higher baseline AMC level and higher baseline MLR level as continuous variables, were both significantly associated with shorter OS (aHR: 2.08, 95% CI 1.21–3.58, p = 0.01, and aHR: 1.27, 95% CI 1.05–1.54, p = 0.01, respectively, Table 3). We further tested whether the laboratory cutpoints associated with irAEs were also associated with OS. There was no significant association between baseline ALC > 2.6 k/μl and OS (aHR: 0.61, 95% CI 0.28–1.36, p = 0.23; Fig. 1A) or between baseline AMC > 0.29 k/μl and OS (aHR: 1.17, 95% CI 0.69–1.99, p = 0.55; Fig. 1D). However, baseline NLR ≤ 5.3, MLR ≤ 0.73, PLT > 145 and PLR ≤ 534 were all associated with longer OS (aHR: 0.68, 95% CI 0.48–0.94, p = 0.02; aHR: 0.43, 95% CI 0.30–0.60, p < 0.001; aHR: 0.48, 95% CI 0.33–0.71, p < 0.001: aHR: 0.48, 95% CI 0.26–0.86, p = 0.015 , respectively, Fig. 1B,C,E,F).

### Association of clinicopathologic features with OS

Patients with personal or family history of AD receiving ICI did not have significantly different OS compared to those without such history (aHR: 1.24, 95% CI 0.85–1.79, p = 0.26; aHR: 0.62, 95% CI 0.25–1.52, p = 0.29, respectively). Similarly, neither the concomitant combination of ICIs nor the history of chronic infection showed a significant association with OS (aHR: 1.28, 95% CI 0.88–1.86, p = 0.19; aHR: 1.29 95% CI 0.81–2.05, p = 0.28, respectively).

## Discussion

Utilizing comprehensive clinicopathologic and laboratory data, this retrospective cohort study details the relationships between specific blood count biomarkers, as well as patient’s health history with the development of irAEs. In our study, development of irAEs was significantly associated with higher baseline ALC (optimal cut-off > 2.6 k/μl), higher baseline AMC (optimal cut-off > 0.29 k/μl), higher baseline platelet count (optimal cut-off > 145 k/μl), lower baseline NLR (optimal cut-off ≤ 5.3), lower baseline MLR (optimal cut-off ≤ 0.73) and lower baseline PLR (optimal cut-off ≤ 534). Additionally, use of concomitant ICI, family and personal history of autoimmune diseases were identified as independent factors associated with irAEs. Notably, while family history has been assumed or implicated as an independent risk factor, our study is one of the first to show this association in a relatively large cohort.

Regarding blood cell count subsets, our findings partially align with prior studies that demonstrated that patients with solid tumors treated with PD-1 inhibitors (nivolumab or pembrolizumab) who had an ALC > 2 k/μl at baseline were at a higher risk of irAEs^17^. Our results demonstrated that ALC > 2.6 k/μl at the time of initiation of ICI was associated with higher odds for irAE development. This finding may suggest that peripheral lymphocytosis may reflect a state of T cell activation that may result not only in enhancement of anti-tumor activity but also higher risk of toxicity. If our hypothesis is validated by future studies, exploration of lymphocyte-directed therapies may be a clinically relevant hypothesis for the management of irAEs that can be explored in clinical trials^17, 18^.

Multiple prior studies have also shown the association between NLR, irAE development and OS^19–21^. In a study of patients with advanced non-small cell lung cancer (NSCLC) treated with anti-PD-(L)1 agents, baseline NLR < 3 was significantly associated with development of irAEs. Another study of patients with NSCLC treated with ICIs also noted an association between NLR < 5 and irAEs. In addition, a different cohort of patients with NSCLC treated with nivolumab showed that pretreatment NLR ≥ 5 was associated with inferior OS. Similar to those findings, we demonstrated that pretreatment NLR ≤ 5.3 was associated with higher odds of irAEs and longer OS. These results suggest that lower NLR might enable a more efficient anti-tumor immune response but also portend a higher risk of immune-related toxicity. This may be due to pro-inflammatory cytokines in the plasma of patients with higher NLR, which may impact the tumor microenvironment and facilitate aggressive tumor behavior^22^. In addition, higher NLR has been associated with an increase in peripheral blood regulatory T cells that can suppress excessive immune responses^23^.

Increased MLR is another potential indicator of systemic inflammation that has been associated with shorter OS in patients with cancer treated with immunotherapy^24^. In our study, we showed that higher MLR and AMC as continuous variables were associated with shorter OS. Type II tumor-associated macrophages from circulating monocytes can suppress the immune reaction against tumor cells^25, 26^. Lower AMC as a continuous variable during ipilimumab treatment in patients with melanoma was associated with favorable outcome^27^. In our study, baseline AMC > 0.29 k/μl did not have a significant impact on OS, but baseline MLR ≤ 0.73 was associated with almost three-fold higher odds of irAEs and also longer OS. Lower MLR ratio may possibly reflect the balance between the favorable role of lymphocytes and unfavorable effect of particular monocyte types in this setting. The literature is still evolving regarding blood-based biomarkers, including platelets, which can be impacted in inflammatory states^19^. Our findings regarding PLR merits further investigation, including validation of prognostic role also based on the identified cut-off value.

In addition to laboratory values, demographic and clinicopathologic features, such as family and/or personal history of AD, may be relevant to the development of IRAEs. There are several reports of familial aggregation of rheumatoid arthritis and/or other autoimmune diseases, including systemic lupus erythematosus, Sj**ö**gren’s syndrome, ankylosing spondylitis, systemic sclerosis, polymyalgia rheumatica, Hashimoto thyroiditis, hypothyroidism, psoriasis, vasculitis or sarcoidosis^28–31^. The presence of diverse autoimmune disorders in family members of patients who developed irAEs may imply potential hereditary susceptibility to autoimmunity.

In a recent study, four defined human leukocyte antigen (HLA) loci most frequently associated with autoimmune diseases were studied in association with development of irAEs in patients with metastatic cancer during ICI therapy. Two HLA risk alleles that predispose to autoimmune diseases (HLADRB1*11:01 and HLA-DQB1*03:01) were found to be associated with occurrence of pruritus or colitis during ICI therapy. However, multicenter large genome-wide association studies in patients with personal and/or family history of autoimmune disease are needed to further assess genetic associations of irAEs^32^.

Given that genetic factors may possibly play a role in irAE development, a detailed family history of AD may be a useful tool in identifying those who may be at higher risk. We did not perform additional analyses regarding the specific types, severity, or the degree of relatives with AD, but this can be the focus of future dedicated studies.

Many studies have shown an increased risk of irAE or AD flare among patients with pre-existing AD or prior irAE^33–35^. In a recent systematic review that studied the use of ICIs in the treatment of patients with cancer and pre-existing AD, exacerbation of pre-existing AD (41%), de novo irAEs (25%), or both (9%) developed in 75% of patients^36^. Initially, patients with pre-existing AD were excluded from many clinical trials of ICIs because of increased risk of development of irAEs^37^. However, patients with well controlled, mild to moderate pre-existing AD may be often treated with ICIs. For patients with metastatic melanoma or NSCLC also with AD requiring no or only low dose immunosuppression, data from retrospective studies suggest that a minority of patients (20–40%) experienced exacerbation of their AD after administration of ICIs, which were relatively manageable^38–40^. In our study, 17% of patients had a pre-existing history of AD and only 3% of patients in our cohort were on immunosuppressive therapy (including steroids) prior to ICI initiation of (Table 1). The increased irAE rate with the use of concomitant ICIs is well described in the literature^1, 41, 42^.

Our study has a number of limitations inherent to the retrospective study nature. First, patients received single agent and/or combination of ICIs for different cancers, indicating major heterogeneity and variability in the patient population, clinical setting, functional status, medical comorbidities, obesity, organ function, treatment duration, prior different lines of therapies, surveillance/monitoring strategies and follow up times. Although our statistical analysis attempted to adjust for differences in several clinicopathologic factors, recall, selection and residual confounding biases are inevitable; therefore, association may not reflect causation. There is also a possibility of misclassification related to data availability and missing/unknown data. For example, charts may not have the most complete family histories of AD so this exposure may be under-represented; we also were not able to capture irAE grade (e.g. based on CTCAE system) due to the retrospective nature of the study. Additionally, we acknowledge that this is a single center study amenable to potential regional influences.

The study has also strengths. Our sample size of 470 diverse patients with a wide variety of tumor types and the systematic strategy for granular data abstraction enabled a solid hypothesis-generating identification of putative factors associated with irAEs. A significant proportion of our results is consistent with previously published data and suggest that blood count subsets could possibly be used in the future, upon further validation, as inexpensive and easily obtained putative biomarkers to help predict the development of irAEs.

To conclude, our findings in this retrospective cohort study support the notion that in patients with cancer prior to initiation of ICI, family or personal history of autoimmune disease, as well as baseline laboratory assessment are significantly associated with IRAEs. Upon further validation, our findings may possibly contribute to more accurate estimation of irAEs and support discussions on future development of prognostic nomograms and models that can be further tested in clinical trials.
